# A novel peroral cholangioscopy of gallbladder carcinosarcoma: a case report

**DOI:** 10.1055/a-2621-2963

**Published:** 2025-07-04

**Authors:** Takumi Onoyama, Taro Yamashita, Takuya Shimosaka, Yuri Sakamoto, Noriyuki Suto, Tsuyoshi Mikamo, Hajime Isomoto

**Affiliations:** 1 Division of Gastroenterology and Nephrology, Faculty of Medicine, Tottori University, Yonago, Japan

Gallbladder carcinosarcoma (GBCS) is extremely rare, accounting for less than 1 % of malignant primary gallbladder tumors with poor prognosis 1 2 3 4 . Due to its rarity, the literature on GBCS is limited, with only approximately 100 cases reported 5 . We herein present a case of GBCS in which the tumor was visualized directly using a novel peroral cholangioscope (eyeMax; Micro-Tech, Nanjing, China).

A 74-year-old female visited a medical institution due to loss of appetite, where a gallbladder mass was detected, and she was admitted to our hospital. Contrast-enhanced abdominal computed tomography showed a mass from the gallbladder neck to the common bile duct (CBD). An enlarged Lymph node with contrast enhancement was observed between the extrahepatic bile duct and gallbladder ( Fig. 1 **a, b** ). The patient also underwent magnetic resonance cholangiopancreatography, which demonstrated a filling defect in the CBD, near the junction of the cystic duct ( Fig. 1 **c, d** ). Endoscopic ultrasonography revealed a hypoechoic lesion in this region ( Fig. 2 **a, b** ). Endoscopic retrograde cholangiopancreatography was performed to confirm the diagnosis of the biliary lesion and revealed a filling defect at the junction of the cystic duct ( Fig. 3 ). Peroral cholangioscopy (POCS) revealed an irregular subepithelial-like lesion with irregularly dilated vessels at the junction of the cystic duct, which was suspected to be a malignant tumor ( Fig. 4 **a, b** and Video 1 ). The histopathological findings of the forceps biopsies from the lesion revealed the diagnosis of carcinosarcoma, which consists of adenocarcinoma, sarcomatous cells, and chondroid matrix ( Fig. 5 ). POCS-guided mapping biopsy also showed that atypical epithelial cells exist in the confluence of intrahepatic bile ducts. The patient preferred best supportive care over aggressive treatment. To the best of our knowledge, this is the first report of the visualization of GBCS using a novel POCS.

**Fig. 1:**
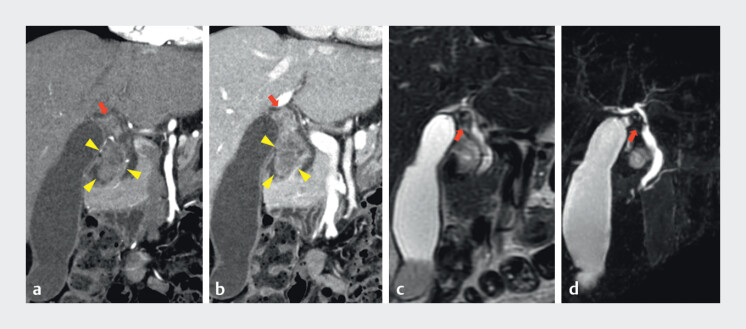
Computed tomography image and magnetic resonance cholangiopancreatography. **a, b** A mass from the gallbladder neck to the extrahepatic bile duct was identified (arrows). Enlarged Lymph node with contrast enhancement was observed between the extrahepatic bile duct and gallbladder (arrowheads). **c, d** Filling defect in the biliary system, from the gallbladder neck to the junction of the cystic duct (arrows). There was no dilation in the common hepatic duct.

**Fig. 2:**
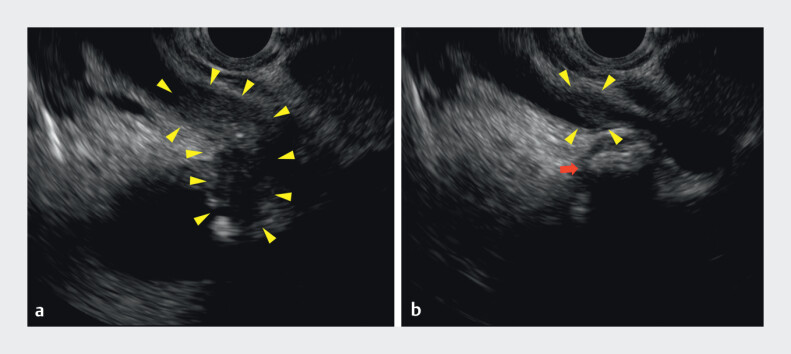
Endoscopic ultrasonography. **a, b** There was an irregular hypoechoic lesion from the gallbladder neck to the extrahepatic bile duct (arrowheads). A gallbladder stone exists near the lesion (arrow).

**Fig. 3:**
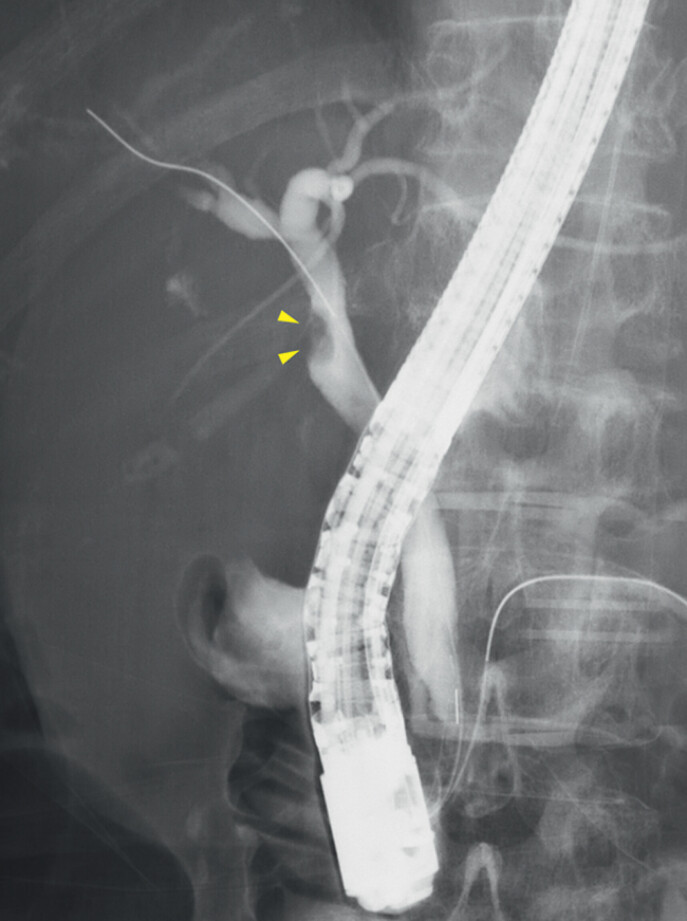
Endoscopic retrograde cholangiopancreatography showing. A filling defect is observed in the common bile duct near the junction of the cystic duct (arrowheads).

**Fig. 4:**
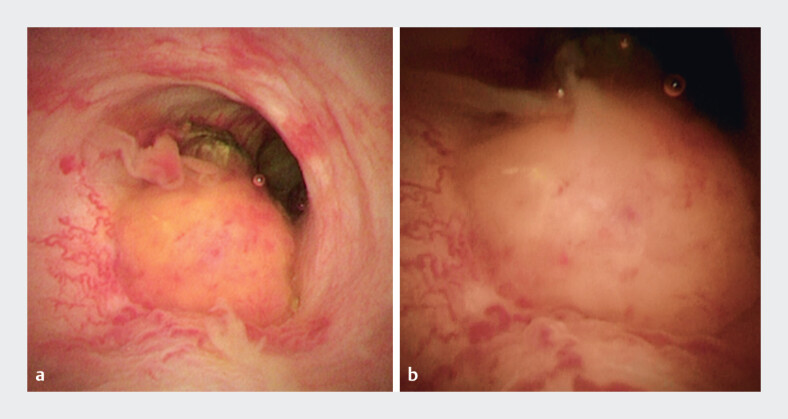
Peroral cholangioscopy. **a, b** There was a subepithelial-like lesion with irregularly dilated vessels at the junction of the cystic duct.

The video shows a case of gallbladder carcinosarcoma in which the tumor was visualized directly using a peroral cholangioscope. It seemed a subepithelial-like lesion with irregularly dilated vessels.Video 1

**Fig. 5:**
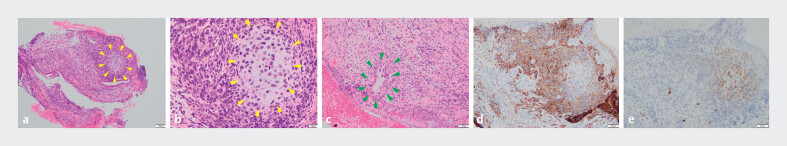
Hematoxylin and eosin staining images of the forceps biopsy sample. **a, b** Chromatin-rich short spindle cells are proliferating diffusely within the stroma. The chondroid matrix was also observed in the stroma (yellow arrowheads). **c** Atypical cells with irregular nuclei and disrupted polarity were proliferating, forming glandular ducts (green arrowheads). The above findings suggested a mixture of adenocarcinoma, sarcomatous cells, and chondroid matrix, and it was diagnosed as carcinosarcoma. **d** Adenocarcinoma and sarcomatous components were positive with immunohistochemical staining for pan-cytokeratin (CK AE1/AE3). **e** The chondroid matrix was immunohistochemically positive for S100.

Endoscopy_UCTN_Code_CCL_1AZ_2AC

## Notes

Endoscopy E-Videos https://eref.thieme.de/e-videos
*E-Videos* is an open access online section of the journal *Endoscopy* , reporting on interesting cases and new techniques in gastroenterological endoscopy. All papers include a high-quality video and are published with a Creative Commons CC-BY license. Endoscopy E-Videos qualify for HINARI discounts and waivers and eligibility is automatically checked during the submission process. We grant 100% waivers to articles whose corresponding authors are based in Group A countries and 50% waivers to those who are based in Group B countries as classified by Research4Life (see: https://www.research4life.org/access/eligibility/ ). This section has its own submission website at https://mc.manuscriptcentral.com/e-videos .

## References

[1] Cao R Jiang H Zhang Y Comparison of carcinosarcoma and adenocarcinoma of the gallbladder: a study based on SEER population for propensity matching and nomogram analysis J Cancer Res Clin Oncol 2023 149 13985 13993 10.1007/s00432-023-05220-0 37543541 PMC11796759

[2] Teng TZJ Chua BQY Shelat VG Carcinosarcoma of gallbladder: A world review World J Clin Oncol 2021 12 1244 1263 10.5306/wjco.v12.i12.1244 35070742 PMC8716988

[3] Okabayashi T Shima Y Iwata J Surgical outcomes for 131 cases of carcinosarcoma of the hepatobiliary tract J Gastroenterol 2014 49 982 991 10.1007/s00535-013-0882-2 24162331

[4] Okabayashi T Sun ZL Montgomery RA Surgical outcome of carcinosarcoma of the gall bladder: A review World J Gastroenterol 2009 15 4877 4882 19842216 10.3748/wjg.15.4877 PMC2764963

[5] Mansour S Derkach E Abergil V Carcinosarcoma of the Gallbladder: A Rare Tumor World J Oncol 2022 13 103 106 10.14740/wjon1495 35837320 PMC9239502

